# How do medical students make sense of internal and external feedback to enhance their Dutch communication skills?

**DOI:** 10.1186/s12909-025-06845-0

**Published:** 2025-02-17

**Authors:** Hao Yu, Zhien Li, S. Eleonore Köhler, Jeroen J. G. van Merriënboer, Maryam Asoodar

**Affiliations:** 1 School of Health Professions Education, Faculty of Health, Medicine & Life sciences, Maastricht University, Maastricht, the Netherlands; 2 Department of Anatomy and Embryology, Maastricht University, Maastricht, The Netherlands

**Keywords:** Medical Dutch, Communication skills, Simulated patient consultations, Internal feedback, External feedback, Reflective practice, Self-regulation, Second Language learning

## Abstract

**Background:**

Feedback is crucial in medical education for developing communication skills and fostering comprehensive learning. Despite its importance, medical students often face challenges in effectively leveraging feedback. This study investigates how students perceive and make sense of internal and external feedback in a 2nd language (L2) medical Dutch course.

**Methods:**

Sixteen third-year international medical students (mean age = 23) participated in a medical Dutch course that included six structured sessions. Each session encompassed a briefing, simulated patient consultations (SPCs), and a debriefing. The curriculum integrated internal feedback from self-reflections and external feedback from peers, teachers, and simulated patients. Data were gathered through a students’ feedback perception survey and semi-structured interviews and analyzed via inductive thematic analysis.

**Results:**

Survey data indicated a trend where the preference, satisfaction, and trust in external feedback were higher than those for internal feedback. However, both types of feedback were regarded as equally effective in facilitating learning progress. Through thematic analysis, we identified five crucial themes that show how students perceive and make sense of various forms of feedback: proactive engagement with feedback, critically analyzing and utilizing the exchange in dialogues and discussions, self-reflection and progress tracking, value from diverse perspectives, and moment-specific and actionable feedback.

**Conclusions:**

This study emphasizes the vital roles of internal and external feedback in enhancing medical Dutch communication skills among medical students. Internal feedback encourages self-reflection and growth, essential for complex medical communications, while external feedback provides clear, specific and supportive guidance and experience from teachers, simulated patients and peers. These feedback mechanisms together improve students’ skills in medical Dutch communication, leading to better doctor-patient interactions. Future research should focus on adapting these feedback strategies across diverse educational settings to further support the development of medical L2 communication skills in global medical contexts.

**Supplementary Information:**

The online version contains supplementary material available at 10.1186/s12909-025-06845-0.

## Introduction

Feedback plays a vital role in the education of medical students, aiding the development of their communication skills and overall learning [1]. However, students often struggle to recognize how the feedback they received from various sources enhances their learning process [2–4]. Researchers have investigated how students make sense of feedback, focusing specifically on their interpretation and understanding of the feedback they receive. For instance, researchers examine feedback from the students’ perspective, such as how students appreciate and prefer certain types of feedback in their learning [5, 6]; their satisfaction with the feedback they receive [7, 8], the extent to which they trust the feedback [5, 6]; and whether the feedback they receive positively influences their learning outcomes [4]. Despite these efforts, research on communication skills in a medical context still lacks depth in exploring how medical students perceive various forms of feedback throughout their learning process.

Making sense of feedback refers to the internal process students undergo as they construe personal meanings from external information, ultimately aiming to translate those meanings into action [9, 10]. Existing research on making sense of feedback has explored several distinct theories. van der Kleij [11] investigated the process of “unpacking” feedback, where learners decipher the task requirements, standards, and expectations conveyed through feedback. Esterhazy and Damşa [12] highlighted a meaning-making process that emphasizes the critical role of feedback in helping learners understand new information and link it to their existing knowledge. This process includes making messages clear and specific, aligning feedback with individual goals, and addressing the emotional reactions that feedback typically provokes, which can significantly affect how it is received etc [13, 14].

Learning to make sense of feedback from various sources is essential for medical students, who must adeptly navigate the complexities of clinical environments in their future careers. Recent findings highlight the critical need for this skill, as students often struggle to accurately synthesize feedback from multiple sources to develop a comprehensive understanding of their environment [15]. Feedback can vary based on its sources and can serve different functions. Generally, feedback can be classified into two primary categories: internal and external. Internal feedback originates from within an individual, while external feedback comes from others [16]. Internal feedback arises from an individual’s active self-reflection and is seen as the insights students gain when they assess their current knowledge and abilities against a specific standard [16, 17]. External feedback, on the other hand, provides observable outcomes and progress, assisting students in identifying areas for improvement [18, 19]. Narciss et al., [18] found that combining internal and external feedback in learning abstractive knowledge can be more beneficial than relying solely on one type. Nicol [17] examined how learners leverage external feedback to generate internal reflections and insights. However, from the students’ perspective, it remains unclear how they make sense of internal and external feedback to support the development of their medical Dutch communication skills.

This study aims to answer the following research question: How do medical students perceive and make sense of internal and external feedback in their second language (L2) learning for medical Dutch communications during simulated patient consultations (SPCs)?

## Methods

This research employed a mixed-methods approach to enable a comprehensive and in-depth investigation. Data were collected through a feedback perception survey and follow-up interviews. The data were reported in accordance with the Consolidated Criteria for Reporting Qualitative Research (COREQ) checklist [20].

### Research team and reflexivity

#### Personal characteristics and research group

To understand how students perceive and make sense of internal and external feedback, researchers HY, JvM, and MA developed the interview guideline. All three researchers were well-versed in the context of feedback usage in medical communication. As one of the course designers, MA had the opportunity to meet all participants during the first few sessions of the course. However, she did not play a direct role in recruiting participants. The interviews were conducted by HY through Microsoft Teams, an online meeting platform. HY was the interviewer and was extending invitations to students to participate as interviewees.

We have ensured a variety of perspectives for ongoing reflection on the data within our team. HY and ZL are fourth year and second-year PhD candidates in medical education, respectively. MA, JvM, and SEK are senior staff and researchers in the field of medical education. MA and JvM possess extensive experience in research on feedback and medical communication, as well as in course design. SEK has extensive knowledge and experience in teaching.

### Study design

#### Context

**Fig. 1:**
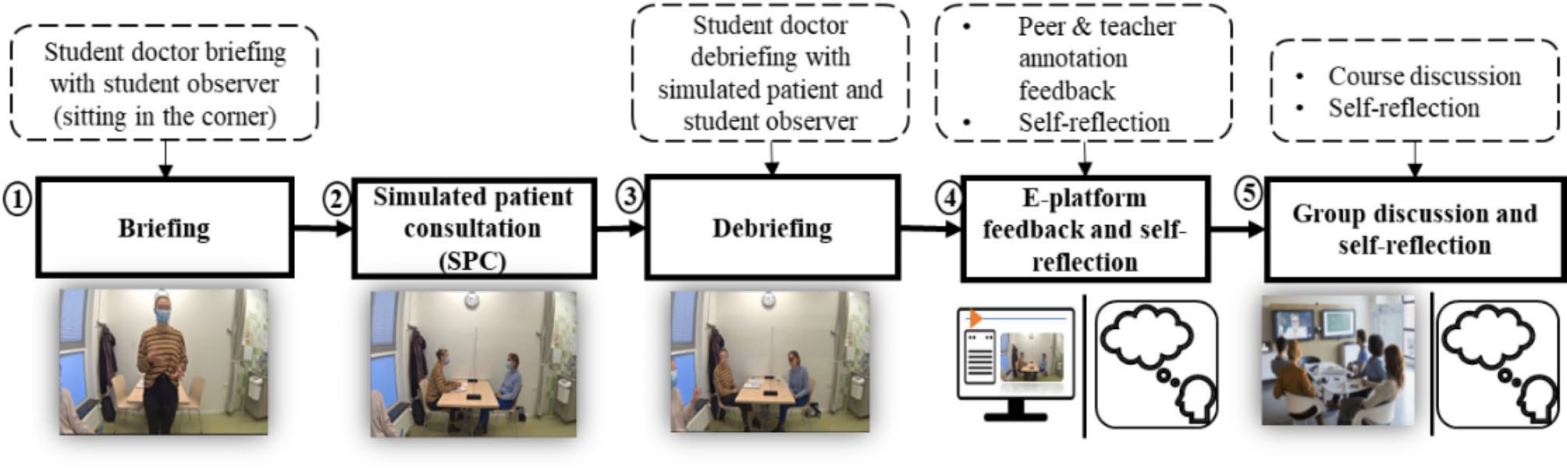
Feedback process centered around simulated patient consultation

This investigation focuses on a medical Dutch course designed for international students who will be studying medicine at Maastricht University, and Dutch is their second or third language. The course aims to enhance Dutch medical communication skills through simulated patient consultations (SPC). This research examines how the course prepares medical students for real interactions with patients by improving their medical communication skills in Dutch through SPCs. The SPCs take place in the SkillsLab designed to mirror a real medical consultation room, complete with actors trained to simulate patients with specific medical histories, thereby offering a consistent and structured learning environment. In each session, one student is the doctor, actively engaging in a consultation with a simulated patient, while another student (a peer) acts as an observer, and silently and critically monitors the student-doctor’s performance to provide feedback after the consultation. Feedback is incorporated into the SPC process as a pivotal element, as outlined in Fig. 1.

Figure 1 shows how internal and external feedback are integrated into the SPC design. In **Step 1**,** ‘Briefing**,**’** the student-doctor explains their specific learning goals and the strategies for achieving those learning goals. This process is dominated by internal feedback due to its self-reflective nature. In **Step 2**,** ‘Simulated Patient Consultation (SPC)’** the student-doctor conducts a medical consultation in Dutch with a simulated patient, forming the core of the SPC design. In **Step 3**,** ‘Debriefing’**, the student-doctor actively seeks external feedback from the simulated patient and the observer, a peer, who is closely observing the consultation from a close distance within the room. Subsequently, the recording of the SPC is uploaded to a digital platform (CAE Learning Space). In **Step 4**,** ‘E-platform and self-reflection’**, peers and the course teacher who were not present during the SPC, can now watch the recording and provide annotated feedback on the recorded videos for the student-doctor. This feedback highlights strengths and areas of improvement in the medical Dutch communication skills. The student-doctor has to reflect on his/her own performance and also provide annotated feedback. The final step, **Step 5**,** ‘Group discussion and self-reflection’**, occurs in a group setting where student-doctor, peers, and the teacher collaboratively discuss the performance of the student doctor and the annotated feedback on the video in a face-to-face session. This session provides students with opportunities to either refine and establish new learning goals by discussing with others or by themselves. Six rounds of SPC were planned for each student, tailored to the course chapters, such as diabetes, autoimmune diseases, respiratory diseases, heart disease, oncology, and neurology.

#### Theoretical framework

The study’s conceptual framework is informed by van der Kleij [11] and Esterhazy and Damşa [12], who have contributed significantly to our understanding of the process of making sense of feedback. To enhance our comprehension of students’ perceptions, based on previous investigation in the literature that focused on making sense of feedback from students’ interpretation and understanding, four critical dimensions were used to measure: feedback preference, satisfaction, trustworthiness, and the facilitative effect of feedback. These dimensions enable us to comparatively understand how students perceive and make sense of both internal and external feedback.

#### Research method

Our study employed a mixed-methods design, utilizing students’ perception surveys and semi-structured interviews to investigate how students make sense of internal and external feedback.

#### Sample

Sixteen third-year international medical students (man = 3, woman = 13; mean age = 23), voluntarily consented to participating in the study. Upon their completion of the study, each participant was compensated with a voucher for their time. All participants held a Common European Framework of Reference for Languages (CEFR) Level B2 proficiency in Dutch.

We collected demographic data on the participants, including age, gender, and prior language learning experience (Table 1).

**Table 1:** Demographic data of the 16 participants

Demographics	Value
Age (mean)	23
Gender	
Man	3
Woman	13
Common European Framework of Reference for Langauge (CEFR) Level	B2

Ethical approval for the study was granted by the Faculty of Health, Medicine, and Life Sciences Ethics Committee (reference number FHML-REC/2021/104). Participations were voluntary and the students involved signed consent forms after receiving information on the study.

### Settings

#### Data collection

During the COVID-19 pandemic, H.Y. conducted individual, semi-structured interviews in online meetings, lasting between 30 and 60 min, to assess students’ perceptions regarding the processing and sense-making of internal and external feedback.

### Feedback perception survey

The survey (Supplementary File 1) consists of 8 items structured into four primary sections: feedback preference, feedback satisfaction, feedback trustworthiness, and the progress-facilitating effect of internal and external feedback on learning medical Dutch communication, these items serve as complement idea to the interviews. The survey was validated for content by consulting two educational scientists familiar with our target population. They reviewed the survey to ensure the items were clear, understandable, and relevant. Their feedback was incorporated to refine the final version of the survey. Students answered the survey by rating from Strongly Disagree [1], Disagree [2], Neutral [3], Agree [4] to Strongly Agree [5] to describe how well a statement fitted their perception. Items of the survey are as follows (Table 1):

- *I prefer reflecting on my performance for learning.*
- *I prefer to rely on the feedback that I receive from others in my learning.*
- *I am satisfied with my own reflections in my learning.*
- *I am satisfied with the feedback that I receive from others to fulfill my learning*
- *I trust that my own reflections help me better with my learning.*
- *I trust that the feedback I receive from others is more influential in my learning.*
- *My own reflection on how I have performed the tasks helps me with making progress in my learning*.
- *The feedback that I receive from others helps me make progress in my learning*.

#### Semi-structured interview

The online recordings were conducted using computer screen capture and were deleted after transcribing the audio tracks into text and anonymizing the data. The anonymized data was managed using Atlas.ti. We developed a set of open-ended questions for a comprehensive interview investigation (See appendix for full interview guideline):

- *What are the key differences you perceive between feedback received from others and feedback you derive from your own reflections?*
- *In what ways do you find that your self-reflection and feedback from others contribute to your understanding of medical Dutch?*
- *How do your own reflections and the feedback you receive from others shape your attitude towards learning medical Dutch?*
- *Can you describe how both your self-reflection and feedback from others have affected your overall experience in the medical Dutch course?*

The interview data underwent inductive analysis, resulting in the identification of five distinct themes, as detailed in the results section.

### Data analysis

Feedback perception survey was descriptively analyzed. For the interview data, two members of the research team (HY, ZL) developed an initial coding framework based on the analysis of four transcripts. Members (HY, ZL, MA) then met repeatedly to interpret and provide feedback to develop and evaluate interview themes and explore theoretical foundations. Theme development was facilitated by identifying theories related to making sense of feedback, such as the concepts of unpacking [11], meaning-making [12], and internal feedback generation [17].

In a continuous analysis, new data were systematically compared to the identified codes. After the 16th interview the research team determined that no new themes were emerging from the final set of transcripts, indicating data saturation. All transcripts were reviewed by members of the research team (HY, ZL, MA), leading to a final coding structure and a document of memos. The team (HY, MA, SEK) then discussed the findings, identifying important themes and the relationships between them.

## Result

Our research question explored how students perceive and make sense of internal and external feedback in an L2 medical Dutch course. The survey examined students’ perceptions of internal and external feedback across four key dimensions: feedback preference, feedback satisfaction, feedback trustworthiness, and the progress-facilitating effect on learning medical Dutch communication skills.

The students scored external feedback higher in all key dimensions, except for the progress-facilitating effect where they scored internal feedback as more effective. Interestingly, both internal and external feedback were rated comparable with respect to their perceived progress-facilitating effect (Table 2). We observed a trend where the preference for and satisfaction with external feedback were higher than those for internal feedback. Students perceived external feedback as more trustworthy than internal feedback. Moreover, students gave high ratings to both internal and external feedback for their effect on facilitating progress in their learning.

**Table 2:** Survey of students’ perceptions of internal and external feedback (*N* = 16)

Dimension	Items	Average (SD)	Strongly disagree	Disagree	Neutral	Agree	Strongly agree
Internal Feedback preference	*I prefer reflecting on my performance for learning.*	3.25 (1.07)	0	4	4	4	4
External Feedback preference	*I prefer to rely on the feedback that I receive from others in my learning.*	3.88 (0.88)	0	0	4	8	4
Internal Feedback satisfaction	*I am satisfied with my own reflections in my learning.*	3.31 (0.98)	0	4	4	5	3
External Feedback satisfaction	*I am satisfied with the feedback that I receive from others to fulfill my learning*	3.87 (0.88)	0	0	6	6	4
Internal Feedback trustworthiness	*I trust that my own reflections help me better with my learning.*	2.75 (0.95)	1	5	7	2	1
External Feedback trustworthiness	*I trust that the feedback I receive from others is more influential in my learning.*	4.06 (0.85)	0	0	4	7	5
Progress-facilitating effect of Internal Feedback	*My own reflection on how I have performed the tasks help me with making progress in my learning*	3.85 (0.87)	0	0	5	7	4
Progress-facilitating effect of External Feedback	*The feedback that I receive from others help me progress in my learning*	3.68 (1,03)	0	2	4	5	5

Notes: Due to low number of participants, we determined that statistical testing would not be effective, therefore we chose to report our questionnaire data descriptively

Using inductive thematic analysis, we identified five themes that illustrate how students interpret feedback in their learning of medical Dutch communication (See Coding Framework in Supplementary material Table 1): proactive engagement with feedback, critically analyzing and utilizing the exchange in dialogues and discussions, self-reflection and progress tracking, value from diverse perspectives, and moment-specific and actionable feedback.

### Proactive engagement with feedback

Students found feedback from their teachers and peers useful and actively sought feedback from them. They highly valued knowing what they needed to fix, discovering how they could improve, and enhance their Dutch communication skills.

“I think the most important part for me really comes from having the opportunity to ask for feedback from the simulated patients and the teachers. If you look at it, having the teacher provide feedback brings in the medical comprehension, especially of the Dutch language, while the simulated patients add the empathy, how they feel about my speaking. This combination gives a clear picture of what I need to get out of the program… it just, the more you ask, the more you learn.” (Student 6).

By seeking and using feedback from others, students took an active role in their learning process and continuously worked towards improvement.

“If we have a consultation and nothing is provided, it’s useful for practice but not for improvement. But I rely heavily on feedback, no matter from others, or only I reflect on my own, I need to set the right learning goals for next time, so I have to know how I am actually doing. (student 6)

Students expressed their eagerness to engage with feedback, noting that having a clear goal to focus on is more motivating than navigating their improvements without direction.

“It helps me feel motivated to know what I need to improve and have a specific goal to be focused on instead of kind of reaching in the dark and not really knowing what there is to focus on.” (Student 7).

### Critically analyzing and utilizing the exchange in dialogues and discussions

The students shared that their frequent engagement in deep discussions with their peers was an enriching experience. Experiencing diverse perspectives and challenging each other’s ideas, led to insights and understandings that they never could have achieved alone.

“I think, like feedback from others kind of plays into feedback that I give myself, because if someone like catch it, even or anyone tells me that I’ve made a mistake, I will listen, but then I would have to think about what the correct way is and make sure that even though I know they’re saying the right thing I have to reflect on what they said, I think the feedback from others combined, it always leads to me self-reflecting, so it does make the whole process faster than just me reflecting by myself and not having feedback.” (student 14).

The students frequently remarked that their peers had a specific ability to notice aspects of their SPC that they themselves had overlooked. They emphasized that this led to a broader understanding of the topic and helped fill in gaps in their knowledge, which they identified as the primary benefit of this peer-to-peer interaction.

“Exchange feedback in discussion is often useful for the things that you aren’t aware of. For example, I use a lot of filler words I don’t notice, in Dutch, I don’t have a lot of confidence in doing so. But if someone tells me that I used those a bit too often, it prevents me from developing an idea of keeping doing it. I often use feedback from others to self-reflect.” (student 5).

Students highlighted that there were instances where feedback was difficult to fully comprehend through a single written exchange. They saw feedback as an interactive and iterative process, requiring multiple rounds of engagement and clarification to ensure that the intended message was fully grasped and understood.

“I think the other students, by being in my groups and classes and interacting, also give feedback, and I learn more. It makes it more effective. I think the important thing is to talk through with others (peers, teachers, and simulated patients) that you learn from, not only to know what I have done well, but also what I have done less well. Where can I improve? And then it really makes you think about the next time I’m doing this. How can I do it differently? How can I structure my consultation differently? How do I approach the questions? Can I ask the questions differently?” (Student 10).

The students reported that they actively sought to build relationships and form bonds with their teachers and peers through open and interactive dialogue. They expressed that the supportive atmosphere they experienced was a direct result of the strong connections they established during their learning.

“Whenever I have a question or need help, I will immediately think of my peers. I think that applies to all of us, and we are always ready to help each other. It’s always natural to talk with the teacher and the simulated patient; also, they are active in providing their thoughts to us. I think the environment is absolutely supportive. I wouldn’t learn so much without having them there.” (Student 11).

### Self-reflection and progress tracking

Students indicated that they have a comprehensive understanding of their learning. They emphasized having a clear vision of their goals and actively seeking solutions to address the challenges they encountered. Furthermore, they highly valued their self-reflection and maintained good control over their learning pace, making steady progress in learning medical Dutch communication.

“I often have a distorted idea of how I think I did compared to how I actually did, so I used to tend to trust others’ opinions more. However, it’s important to recognize that opinions can vary widely, and there’s no one perfect way to approach a situation. For instance, when giving a consultation with simulated patients, everyone will have different feedback, even if you say the exact same things. It pushes me to reflect and reflect, until I feel that I am perfect enough on this matter… I become thoughtful in considering my pace in learning, and good in controlling setting my learning goals…” (student 8).

Students mentioned that they could track their progress and identify precisely where they needed to focus their efforts for further improvement.

“I think I naturally like self-reflection, which is something I’m already doing during consultations. I interpret what’s happening, what’s being said, and how the patient is reacting to my words. I already have a mental image of how I can improve my approach. However, I believe that receiving feedback from others helps me better sift through this information and gives me a clearer picture of what’s most important for me to focus on.” (student 2).

### Value from diverse perspective

Students reported that when their self-reflections matched the feedback they received from others, it was a truly validating moment for them. They gained confidence in formulating their learning goals by observing the consistency of insights from multiple sources, such as teachers, peers, and their own reflections.

“I think that confidence and assurance in speaking helped me in learning. It helps, when having people assure you that it was understandable and that it is okay, so that you can begin to feel that yourself.” (student 15).

Students recognized the importance and value of learning from others, as it helped them identify and correct errors they had been unknowingly repeating.

“I really appreciate my peers because sometimes they point out specific phrases I’m using incorrectly or things I repeatedly do wrong without realizing. This helps me make the necessary changes.” (student 13).

Students also indicated that their language improved with external help, such as Dutch pronunciation and grammar guidance from simulated patients and teachers.

“Since I speak German, I sometimes encounter difficulties when using Dutch due to similarities between the two languages. For example, I might use German-influenced phrases or consistently make the same errors without realizing it. Simulated patient understand Dutch because they are native speakers. I appreciate the feedback that helps correct my language use. They are idealized patients.… The Dutch teachers concentrated more on the language itself, but the simulated patients can really provide insights on everything.” (Student 3).

### Moment-specific and actionable feedback

The students expressed appreciation for the personalized feedback that was provided at the right time, tailored specifically to their needs and actionable, making it easier to apply. This applied to the feedback given right after the SPC session, where the patients shared their experience just when the students needed it, and the feedback from the video annotation tool, provided at the moment it was recorded, highlighting points for improvement.

“The feedback from the simulated patient provides more specific and tangible points that I need to focus on. At the moment you are exchanging your thoughts and actions with others, I think it is easier to remember the feedback from others because the environment helps you remember. For self-reflection, you can quickly tell yourself in your head that you can use that quick feedback.” (student 8).

Students looked forward to the meaningful group discussion, where, for example, peers and the teacher were well-prepared to share their knowledge and offer help.

“When you check the recordings or setting learning goals alone, you are really easy to get judging and overly critical to yourself. I have to say that I like the way we exchange our ideas. During the discussions, it’s not like people talking blablabla… wasting time. Even though some feedback is sugar-coated, most of us really think, really experienced, and really prepare ourselves for the discussion. This makes the discussions moments you always look forward to because you really learn something.” (student 4).

Students emphasized the value of practical experience and high-quality feedback, focusing on actual practice and the actionable feedback from others.

“There is a bigger focus on speaking the language and learning to apply it. Overall, I think it’s effective because when I look back at what I have learned and how others have helped me, I realize I couldn’t have done better. I am thankful for my peers and teachers, who were patient enough to explain details and make it actionable. I also trust them because they recognized my growth in group discussions.” (Student 9).

## Discussion

Our study aimed to investigate how medical students perceive and making sense of internal and external feedback to improve their L2 medical Dutch communication skills. The survey results indicated a trend whereby external feedback was slightly more preferred, satisfying, and trustworthy compared to internal feedback. This trend has been consistently observed across various educational settings. Fernández-Toro and Hurd [21] noted that independent learners reported satisfaction with external feedback from teachers and learning environments. Similarly, Donche et al., [22] found that students preferred adopting feedback from external sources, which they trusted more and used to regulate their own learning. Our findings suggested that external feedback from teachers and simulated patients added substantial value to the students’ knowledge. Conversely, internal feedback was often perceived as subjective and overly critical. This perception was supported by Lee and Schallert [23], who found that students believed they were excessively harsh in judging their own performance after discussions with peers and teachers.

Furthermore, students reported that both internal and external feedback contributed to their learning progress. While internal feedback was often perceived as overly critical, students recognized its importance in facilitating personal growth. In contrast, research has shown that students who preferred external feedback attributed their learning advancement to the confidence and engaging attitude fostered by such feedback [23]. Additionally, Carless [24] emphasized the importance of centering students within the feedback process, advocating for active engagement in generating, processing, and responding to feedback to drive educational progress. Our interviews aimed to explore how students in a medical Dutch course made sense of feedback in order to enhance their medical Dutch communication skills. We identified five key themes that helped us understand this process: proactive engagement with feedback, critically analyzing and utilizing the exchange in dialogues and discussions, self-reflection and progress tracking, value from diverse perspectives, and moment-specific and actionable feedback.

Being proactive has long been considered an effective method for enhancing communication skills [25]. Through the “proactive engagement with feedback” theme, we found that students who maintained a positive and motivated attitude towards their own learning goals, which they had identified, were better prepared to face learning challenges [26]. Hattie and Timperley [27] emphasized the importance of clear objectives in enhancing student outcomes by providing direction and fostering self-regulation. Similarly, the theme of a self-reflection and progress tracking aligned with Sadler [28] assertion that students perform better when they have a comprehensive understanding of what is expected. This comprehensive approach enables students to actively monitor their progress and adjust their learning strategies, echoing findings from Zimmerman [29] who noted that self-regulated learners are more likely to achieve academic success.

The theme of critically analyzing and utilizing the exchange in dialogues and discussions demonstrated how interactive discussions enhanced the learning process, supporting Nicol and Macfarlane-Dick [19] argument that feedback should encourage teacher-student dialogue to promote deeper understanding. This ongoing dialogue, which “unpacks” feedback into manageable units, ensures students understand and directly contributes to performance improvements [13, 30, 31]. The theme of ‘value from diverse perspectives’ emphasized the benefits of receiving feedback from multiple sources, echoing Carless et al., [32] study, which noted that varied feedback can provide unique insights and prevent the reinforcement of misconceptions. This dialogistic feedback process not only corrects errors but also broadens students’ understanding and perspective, which is essential for comprehensive learning.

The proactive nature of the “moment-specific and actionable feedback” theme underscored the importance of timely and relevant feedback, which Shute [13] argued is crucial for effective learning. As observed in the study, immediate feedback enabled students to make quick adjustments and apply lessons in real-time. This aligns with Ambrose et al., [33], who emphasized the impact of timely feedback on student performance. The integration of the theme proactive engagement with feedback within moment-specific and actionable feedback illustrates the cyclical nature of feedback, where students not only anticipated future learning needs but also actively sought information to address them. This demonstrated a continuous engagement with the learning material and objectives, as noted by Winstone and Carless [34].

The interplay between internal and external feedback appeared to play a crucial role in enhancing medical Dutch communication skills among students. This is an essential part of the sense-making process, which involves unpacking the message [12], connecting feedback to interpersonal goals [13], and managing emotional responses [14].

Internal feedback, while often perceived as challenging, prompted self-reflection and personal development, enabling students to critically assess and improve their communication skills. It specifically helped in ensuring communication with clear, specific message, ensuring that feedback was precise and directly related to the tasks at hand, thus aiding students in understanding what aspects of their performance needed adjustment.

External feedback complemented this by providing personalized perspectives, offering reassurance, and constructive input from experienced individuals such as teachers and peers. It connected to the students’ educational and professional goals, making its relevance and implications clear and actionable. Moreover, it played a crucial role in emotional processing by helping manage the emotional responses that feedback often evoked, which can significantly influence how feedback is received and acted upon.

Together, these feedback mechanisms created a supportive learning environment where students could refine their skills based on diverse insights, thus facilitating communication in medical contexts. By leveraging both types of feedback, students seemed to develop a more comprehensive understanding of medical Dutch, which was likely to lead to enhanced practical communication abilities. These observations suggested that each form of feedback holds distinct value, contributing uniquely to the learning process within a medical Dutch simulated patient consultation setting. The integration of clarifying messages, connecting feedback to personalized learning goals, and managing emotional responses ensures that the feedback process is not only informative but also empowering and motivational, driving improvements in learning L2 communication skills.

### Limitations and future directions

Several limitations of this study should be acknowledged. Firstly, the study’s small sample size and single-course design may limit the generalizability of the findings to diverse student populations and learning contexts. Secondly, the study focused exclusively on students’ perceptions and experiences regarding the utilization of internal and external feedback for improving communication skills. While our current observations provide insights into this area, the results would have been strengthened with the inclusion of additional sources of data. Thirdly, differentiating between purely internal feedback and internal feedback that has been consolidated with external information presented a challenge in our analysis. While the current study provides valuable insights, a limitation is the lack of a comparative analysis between feedback delivered in native versus second languages. Future research should consider incorporating this design element to more rigorously investigate the role of language in feedback efficacy.

Educators are encouraged to establish a feedback-rich environment that prioritizes clear learning objectives, fosters ongoing dialogue, and ensures the delivery of timely and relevant feedback. These practices are crucial for enhancing student performance in the short term and developing long-term learning competencies essential for academic and professional success. Incorporating a sense-making process, where feedback is unpacked in clear and specific ways, connected to personalized goals, and emotionally processed, can empower students and deepen their engagement with communication learning settings. By exploring the impacts of these feedback mechanisms and sense-making processes across various educational contexts and student populations, future studies can contribute meaningfully to the optimization of feedback strategies, ultimately leading to better learning outcomes, increased student satisfaction, and improved academic achievement. This, in turn, can have a profound impact on the broader population, as it can help bridge the gap in educational attainment and promote greater equity in academic and professional opportunities.

## Conclusion

Our study provides insight into how medical students perceive and making sense of internal and external feedback to enhance their L2 medical Dutch communication skills. The questionnaire indicated a slight preference for external feedback, which students found more satisfying and trustworthy, aligning with broader educational trends. However, internal feedback, despite being perceived as more critical, was essential for personal growth. The integration of both feedback types fosters a dynamic learning environment, equipping students to adeptly manage the subtleties of medical Dutch. This study suggests that each form of feedback holds distinct value, contributing uniquely to the learning process within a medical Dutch simulated patient consultation setting. The interplay between internal and external feedback plays a crucial role in the sense-making process, involving unpacking the message, connecting feedback to goals, and managing emotional responses. By leveraging both types of feedback, students develop a comprehensive understanding of medical Dutch, leading to enhanced practical communication abilities. Future research should explore how these feedback mechanisms can be adapted and optimized for diverse educational settings to support L2 communication skill development in medical education.

## Electronic supplementary material

Below is the link to the electronic supplementary material.

Supplementary Material 1

Supplementary Material 2

## Data Availability

https://dataverse.nl/dataset.xhtml? persistentId=doi:10.34894/BFYNGV.

## Abbreviations

L2: second language
SPC: Simulated patient consultation
