# Supplementary material for: How do medical students make sense of internal and external feedback to enhance their Dutch communication skills?
Source: BMC Med Educ. 2025 Feb 17;25:256. doi: 10.1186/s12909-025-06845-0 (PMC11834736; doi:10.1186/s12909-025-06845-0)
Supplement: Supplementary file 1 — Supplementary Material 1 [file 12909_2025_6845_MOESM1_ESM.docx]

Students answer the survey by rating from Strongly Disagree (1), Disagree (2), Neutral (3), Agree (4) to Strongly Agree (5) to describe how well a statement fitted their perception.

| Dimension | Items | Rating scales |
| --- | --- | --- |
| Internal Feedback preference | *I prefer reflecting on my performance for learning.* | Strongly Disagree (1), Disagree (2),  Neutral (3), Agree (4), Strongly Agree (5) |
| External Feedback preference | *I prefer to rely on the feedback that I receive from others in my learning.* | Strongly Disagree (1), Disagree (2),  Neutral (3), Agree (4), Strongly Agree (5) |
| Internal Feedback satisfaction | *I am satisfied with my own reflections in my learning.* | Strongly Disagree (1), Disagree (2),  Neutral (3), Agree (4), Strongly Agree (5) |
| External Feedback satisfaction | *I am satisfied with the feedback that I receive from others to fulfill my learning* | Strongly Disagree (1), Disagree (2),  Neutral (3), Agree (4), Strongly Agree (5) |
| Internal Feedback trustworthiness | *I trust that my own reflections help me better with my learning.* | Strongly Disagree (1), Disagree (2),  Neutral (3), Agree (4), Strongly Agree (5) |
| External Feedback trustworthiness | *I trust that the feedback I receive from others is more influential in my learning.* | Strongly Disagree (1), Disagree (2),  Neutral (3), Agree (4), Strongly Agree (5) |
| Progress-facilitating effect of Internal Feedback | *My own reflection on how I have performed the tasks help me with making progress in my learning* | Strongly Disagree (1), Disagree (2),  Neutral (3), Agree (4), Strongly Agree (5) |
| Progress-facilitating effect of External Feedback | *The feedback that I receive from others help me progress in my learning* | Strongly Disagree (1), Disagree (2),  Neutral (3), Agree (4), Strongly Agree (5) |
