# Supplementary material for: How do medical students make sense of internal and external feedback to enhance their Dutch communication skills?
Source: BMC Med Educ. 2025 Feb 17;25:256. doi: 10.1186/s12909-025-06845-0 (PMC11834736; doi:10.1186/s12909-025-06845-0)
Supplement: Supplementary file 2 — Supplementary Material 2 [file 12909_2025_6845_MOESM2_ESM.docx]

Supplementary Table 3. Coding framework.

| **Theme** | **Subtheme** | **Definition** | **Explanation** |
| --- | --- | --- | --- |
| Proactive Engagement with Feedback | Valuing Feedback | Recognizing the importance of feedback as a tool for improvement. | Students understand that feedback from teachers and peers is crucial for enhancing their Dutch communication skills. They appreciate the dual perspectives of medical comprehension from teachers and empathy from simulated patients, which together provide a comprehensive view of their performance. |
|  | Active Engagement | Proactively seeking feedback and incorporating it into the learning process. | Students actively ask for feedback and use it to set learning goals. They believe that continuous feedback is essential for improvement, as it helps them understand their current performance level and areas needing enhancement. |
| Critically Analyzing and Utilizing the Exchange in Dialogues and Discussions | Collaborative Learning | Engaging in discussions to gain diverse perspectives and insights. | Students find value in deep discussions with peers, which expose them to different viewpoints and challenge their ideas. This collaborative environment leads to a broader understanding of the subject matter and helps fill gaps in knowledge. |
|  | Iterative Feedback Process | Viewing feedback as an ongoing, interactive process. | Students recognize that feedback is not a one-time event but requires multiple rounds of engagement and clarification. This iterative approach ensures that they fully grasp and apply the feedback they receive. |
| Self-Reflection and Progress Tracking | Self-Reflection and Goal Setting | Reflecting on one's performance and setting clear learning goals. | Students maintain a comprehensive understanding of their learning progress. They engage in self-reflection to identify areas for improvement and set specific goals, which helps them control their learning pace and make steady progress. |
|  | Tracking Progress | Monitoring one's development and adjusting efforts accordingly. | Students can track their progress and pinpoint areas that require further focus. This awareness allows them to adjust their learning strategies and efforts to achieve their goals more effectively. |
| Value from Diverse Perspectives | Validation and Confidence | Gaining confidence through consistent feedback from multiple sources. | When students' self-reflections align with feedback from teachers, peers, and simulated patients, they feel validated and confident in their learning goals. This consistency reinforces their understanding and helps them trust their progress. |
|  | Error Identification and Correction | Identifying and correcting mistakes with the help of diverse feedback. | Students appreciate the ability of others to point out errors they may not have noticed, such as incorrect phrases or language habits. This feedback is crucial for correcting mistakes and improving their Dutch language skills. |
| Moment-specific and Actionable Feedback | Timely Feedback | Receiving feedback at the right moment to enhance learning. | Students value feedback provided immediately after a session or at relevant moments, as it is easier to remember and apply. This timely feedback helps them make quick adjustments and reinforces their learning. |
|  | Practical Application | Using feedback to make tangible improvements in practice. | Students emphasize the importance of actionable feedback that they can apply directly to their practice. This focus on practical application ensures that they can effectively use the feedback to enhance their Dutch communication skills. |
